# Effects of conservative treatment strategies for iliotibial band syndrome on pain and function in runners: a systematic review

**DOI:** 10.3389/fspor.2024.1386456

**Published:** 2024-08-23

**Authors:** Alberto Sanchez-Alvarado, Chaitrali Bokil, Michael Cassel, Tilman Engel

**Affiliations:** ^1^ Sports Medicine and Sports Orthopaedics, University Outpatient Clinic, University of Potsdam, Potsdam, Germany; ^2^ Institute of Interdisciplinary Exercise Science and Sports Medicine, MSH Medical School Hamburg, Hamburg, Germany

**Keywords:** running-related injury, muscle training, rehabilitation, non-surgical, physical therapy

## Abstract

**Introduction:**

This systematic review summarizes the efficacy of conservative treatment strategies on pain and function in runners with iliotibial band syndrome (ITBS), a prevalent running injury constituting about 10% of all running-related injuries. The multifactorial nature of ITBS necessitates diverse treatment approaches; yet, a consensus on an optimal conservative regimen remains unreported. This review seeks to update and expand upon existing literature with recent rehabilitative approaches.

**Methods:**

A systematic search was conducted in Medline, Web of Science, and CINHAL databases, from inception to June 31, 2024. Inclusion criteria were: (1) reporting of conservative treatments for ITBS in adult runners and (2) pain and function defined as main outcome parameters. The methodological quality was evaluated using the NIH Quality Assessment Tool.

**Results:**

Thirteen out of 616 records met the inclusion criteria (201 participants), including five randomized controlled trials, one case-control study, one pre-test post-test study, and six case studies. Different active and passive treatment strategies were applied as single (five studies) or combined (eight studies) treatments. The average methodological quality was deemed good. Large between-study heterogeneity was present, impeding a meta-analysis to be performed. Hip abductor strengthening (HAS) exercise emerged as a common strategy. The intervention effects on pain reduction ranged from 27% to 100%, and functional improvement from 10% to 57%, over 2 to 8 weeks.

**Conclusion:**

A conservative treatment approach incorporating HAS exercises, possibly augmented by shockwave or manual therapy, is effective for mitigating pain and enhancing function in ITBS-afflicted runners. Finally, the potential of emerging strategies like gait retraining requires further exploration through rigorous trials and comprehensive evidence. Addressing these gaps could refine ITBS management, enhancing treatment outcomes and facilitating runners’ return to sport.

## 1 Introduction

The iliotibial band syndrome (ITBS) is considered the second most common knee pain in runners, after patellofemoral pain syndrome, and accounts for approximately one-tenth of all running injuries (1, 2). Traditionally it was proposed that ITBS is caused by excessive friction between the distal portion of the iliotibial band as it moves over the lateral femoral epicondyle during repeated flexion and extension of the knee (3, 4). However, contemporary theories suggest impingement of the iliotibial band against the lateral femoral epicondyle as the primary cause, leading to pain and functional impairments in affected runners (5, 6). According to research, impingement of the iliotibial band can arise from impairments in neuromuscular function like altered muscle activation patterns, muscle weakness, and decreased proprioception (7, 8). This can lead to compensatory alterations in running patterns which cause increased stress on the iliotibial band thereby contributing to pain in runners with ITBS.

Similarly, running biomechanics might play a role in the development of ITBS. However, a recent systematic review concerning biomechanical risk factors in distance runners found that despite there being several variables identified as biomechanical risk factors in the literature, the level of evidence associated with them ranges from very limited evidence to conflicting evidence (9). Among these variables, the peak hip adduction angle was found with conflicting evidence; increased ITB strain, increased ITB strain rate, and increased femur external rotation were found with very limited evidence; and inconsistent evidence was for less knee flexion angle at foot strike, increased peak knee adduction angle, and peak knee internal rotation angle (9).

Clinically, ITBS presents as a sharp or burning pain on the lateral aspect of the knee, typically occurring between 20° and 30° of knee flexion (10, 11). This pain characteristically increases during running and is associated with reduced function presented as decreased hip and knee range of motion, decreased muscle strength, decreased running speed and distance, and difficulty in daily activities involving repeated knee flexion movements (7, 10).

The ITBS is an overuse injury with an incidence of about 12% of all running-related injuries (12–14). The etiology of ITBS is multifactorial involving intrinsic factors such as joint biomechanics and extrinsic factors like improper training, increase in running mileage, hill running, and, improper footwear (2, 10, 12, 13).

ITBS is typically managed using conservative methods with surgical interventions indicated in refractory cases where conservative management is ineffective (14). The commonly used conservative treatment strategies include anti-inflammatory drugs, exercises, and physiotherapeutic interventions like manual therapy and electrotherapy (17–21). Conservative treatments of ITBS are preferred over surgical methods, however, the lack of standardization and limited evidence in randomized controlled trials highlights the discrepancies between the different reported options (15). Additionally, the variability in the study designs, method, participants' characteristics, and even definitions of injury and risk factors hinders the generalization of suitable strategies for the treatment of ITBS.

Despite the recommendation for a multifaceted treatment strategy for ITBS, the recovery rate, with only 44% of runners returning to their sport after 6–8 weeks of conservative therapy, highlights the complex interplay between biomechanics and other factors in running-related injuries (6, 9, 21–23). The introduction of innovative treatment modalities, such as gait retraining, represents a shift towards addressing not just the symptoms but the underlying abnormal biomechanics and neuromuscular control issues associated with ITBS (16). This shift highlights the growing need for a comprehensive and multimodal approach to effectively manage ITBS in runners. However, the efficacy of these novel strategies, particularly in terms of their long-term benefits, remains to be validated.

The literature on this topic, limited by its age and the scope of the studies, fails to reflect the advancements in treatment methodologies that have emerged from recent research (6, 17). Moreover, the existing reviews rarely focus exclusively on runners, a critical gap given the specificity of ITBS to this population (17–19). Additionally, there has yet to be a consensus on an effective treatment protocol that specifically addresses both pain and functional improvement in runners suffering from ITBS (9, 17, 18). In light of these gaps, this review aims to systematically assess the available evidence for the effects of conservative treatment strategies on pain and function in runners with ITBS.

## 2 Methods

This systematic review was conducted in compliance with the PRISMA (Preferred Reporting Items for Systematic Reviews and Meta-Analyses) checklist (20). The systematic review was not registered before its commencement. In the context of this review, conservative treatments refer to non-surgical methodologies.

### 2.1 Search strategy

Reports were systematically searched in three electronic databases [Medline (via PubMed), Web of Science, and CINHAL] from inception to July 31, 2024. A comprehensive search strategy was used for a variety of terms related to ITBS and running, using Boolean operators to capture the broadest spectrum of relevant literature: (iliotibial band OR ITB OR ITBS OR ITBP OR ITBFS OR iliotibial band strain OR iliotibial track OR iliotibial) AND (runners OR run OR running OR triathlete) AND (treatment OR management OR therapy OR rehabilitation OR physio* OR intervention OR exercise OR physical therapy OR gait OR gait retraining OR gait training OR gait modification).

### 2.2 Eligibility criteria

The PICOS (Population, Intervention, Comparison, Outcome, and Study design) framework was employed as part of the inclusion criteria for selecting articles in the systematic review. English-language original research studies across different research designs: randomized control trials (RCT), clinical trials, cross-sectional studies, case-control studies, and case studies, focusing on conservative treatments of ITBS in adult runners (≥ 18 years of age) were included. For inclusion studies had to assess pain and/or lower extremity function, without limitations on ITBS severity or laterality (unilateral or bilateral). Studies with non-runners populations, other knee injuries, and surgical interventions were excluded. In addition to the systematic database search, the reference lists of all included articles were reviewed to identify any additional relevant studies. This strategy was employed to ensure a comprehensive collection of data and to mitigate the risk of missing pertinent literature.

### 2.3 Data extraction process

All retrieved studies were uploaded to Rayyan QCRI, a systematic review screening software, for the screening process (21, 22). Duplicates were removed and the potential studies were assessed for inclusion. The literature screening of the title and abstract was independently executed by two researchers (CB and AS). Title and abstract screening were performed and studies against the inclusion criteria were eliminated. Subsequently, a full-text review was carried out and studies that did not meet the inclusion criteria were excluded. A final list of studies eligible for inclusion was compiled. Any discrepancy between the reviewers (CB and AS) was resolved through a consensus meeting, with a third independent reviewer (TE) available in case of disagreement.

### 2.4 Data extraction and synthesis

Data extracted encompassed study specifics: author(s) and year of publication, study design, study population and demographics, outcomes (pain and function), treatment strategies, treatment duration, and follow-up. Extracted data was compiled into a predefined spreadsheet. The studies were categorized into combined treatment studies (utilizing more than one treatment strategy) and single treatment studies (using only one treatment strategy). Then, the individual treatment strategies were classified as active or passive strategies. Active strategies were defined as interventions that were actively performed by the participants. Passive treatment strategies were defined as interventions delivered by health professionals.

To summarize and compare pain outcomes, the values from the Numeric Pain Rating Scale (NRPS), ranging from 0 to 10, were converted to a scale from 0 to 100 (by multiplication). This allowed a standard for visualization of the data in the figures. Finally, the overall change score for pain and function outcomes of each study was calculated in percentage between the baseline and the last measurement post-intervention.

### 2.5 Risk of bias and quality assessment

The NIH Quality Assessment Tool facilitated the evaluation of methodological integrity across study types (23). The evaluation was assessed by one reviewer (CB). For each article type, the assessment was based on the respective number of provided items (RCTs: 14 items, pre-post studies with no control group case-control studies: 12 items, case series: 9 items). To make comparisons among the different study types, the total score of each included study was calculated and expressed as a percentage, ranging from 0% to 100%. Then, these were categorized as poor (0%–25%), fair (25%–50%), good (50%–75%), or excellent (75%–100%) (24). The overall risk of bias was assessed solely on the study type (RCTs were deemed with lesser risk than case-control studies, and case series), and the amount of participants included in each treatment option.

## 3 Results

**Figure 1:**
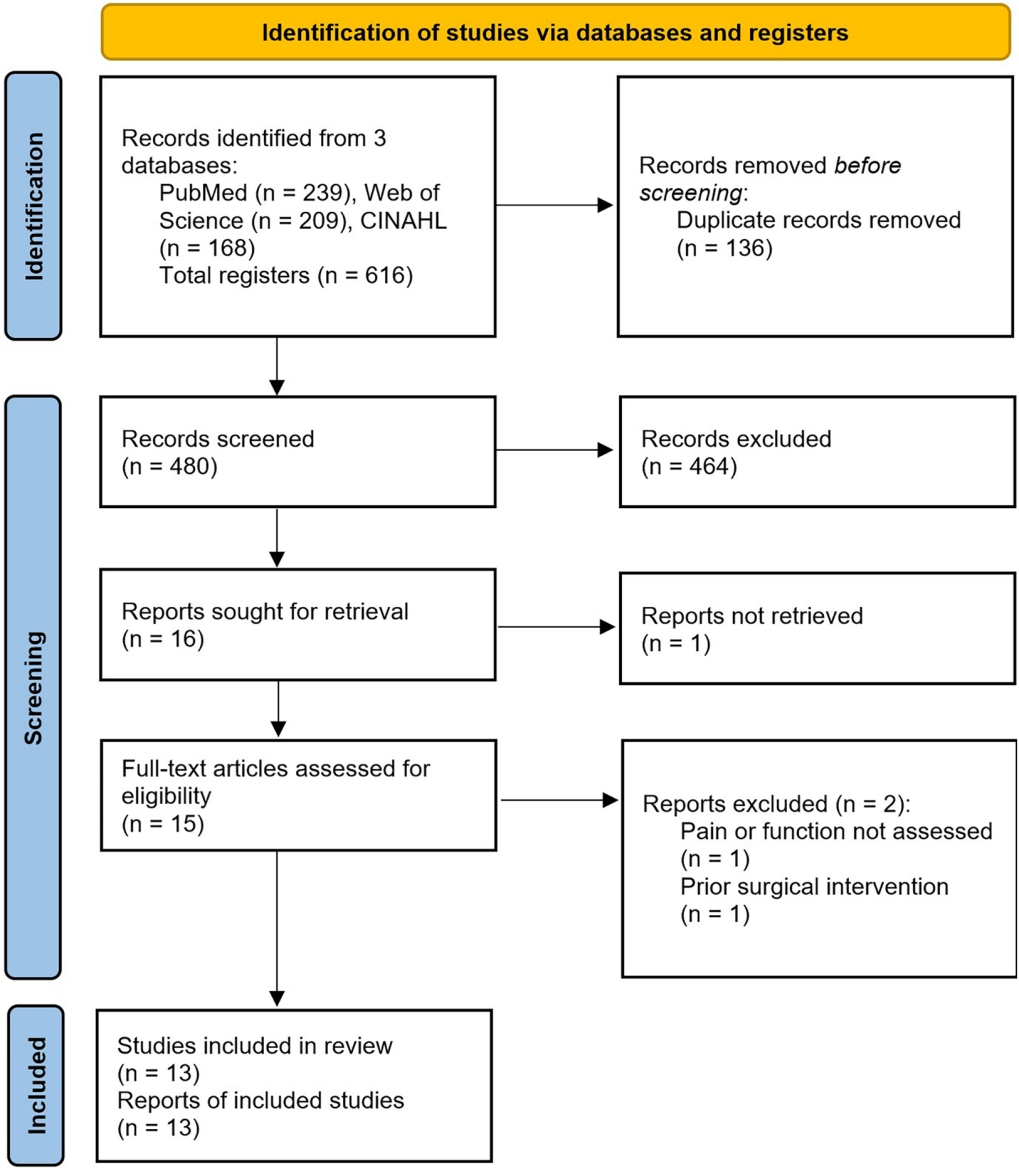
PRISMA flow diagram.

The initial database search resulted in a total of 616 reports. Thirteen studies met the inclusion criteria and were included in this systematic review (see Figure 1). Five were RCTs, one was a case-control study, one was a pre-test post-test study, and six were case series and case reports. In total, the studies included 201 runners with ITBS. The age of the participants ranged from 18 to 60 years, with 60% of them being female (as provided in twelve studies).

The reviewed studies employed various conservative treatment strategies, categorized into active strategies comprising hip abductor strengthening (HAS) exercises, stretching exercises, and gait retraining; and passive strategies which included manual therapy techniques, electrotherapeutic modalities, and dry needling. Overall, the studies used either a single treatment (5 studies) or a combined treatment approach (8 studies).

**Figure 2:**
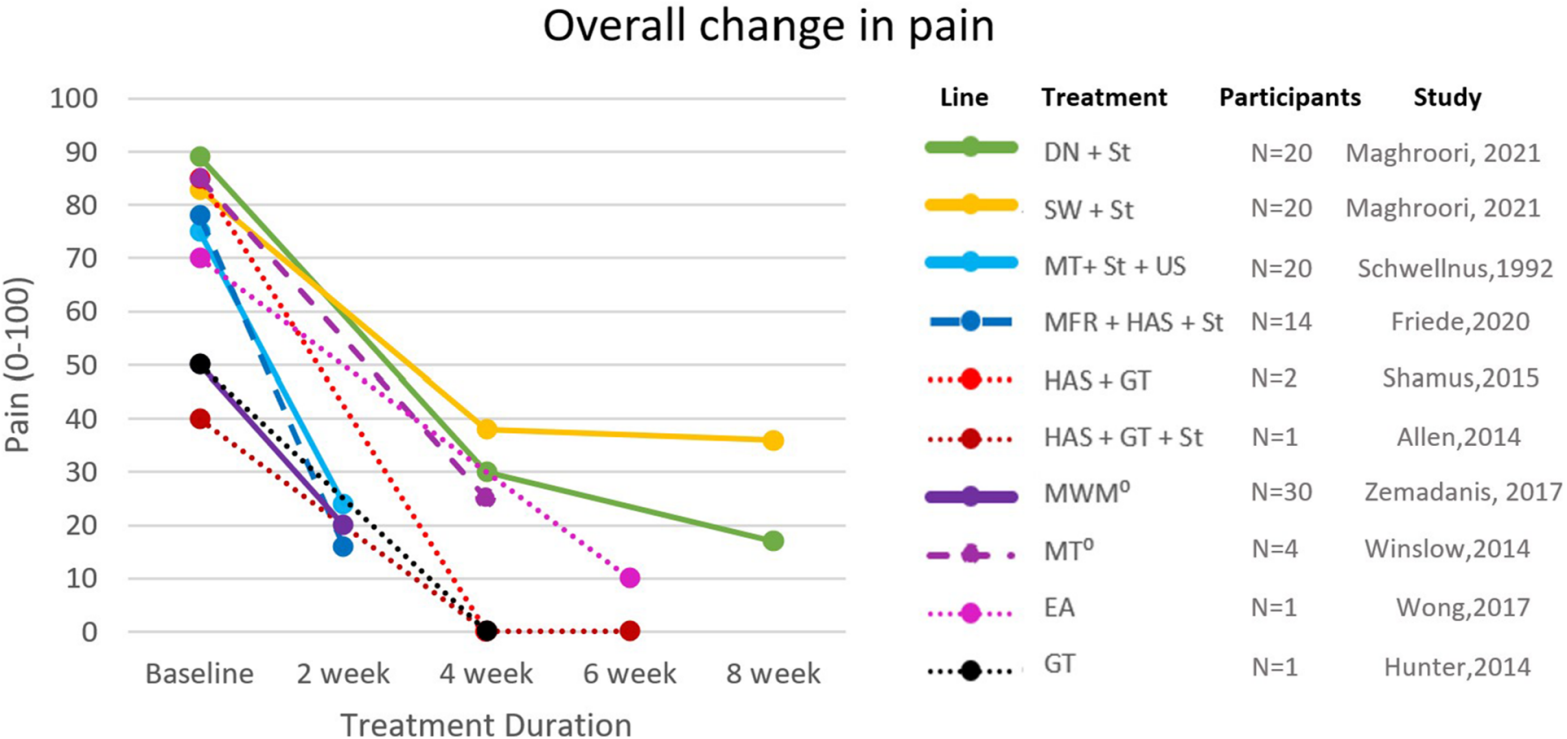
Changes in pain scores across all included studies. Solid lines: RCTs; dashed lines: case-control and case series; dotted lines: case reports; ⁰: values converted from NRPS to VAS; DN, dry needling; EA, electroacupuncture; GT, gait retraining; HAS, hip abductor strengthening; MFR, myofascial release; MT, manual therapy; MWM, movement-with-mobilization; St, stretching; SW, shockwave; US, ultrasound.

**Figure 3:**
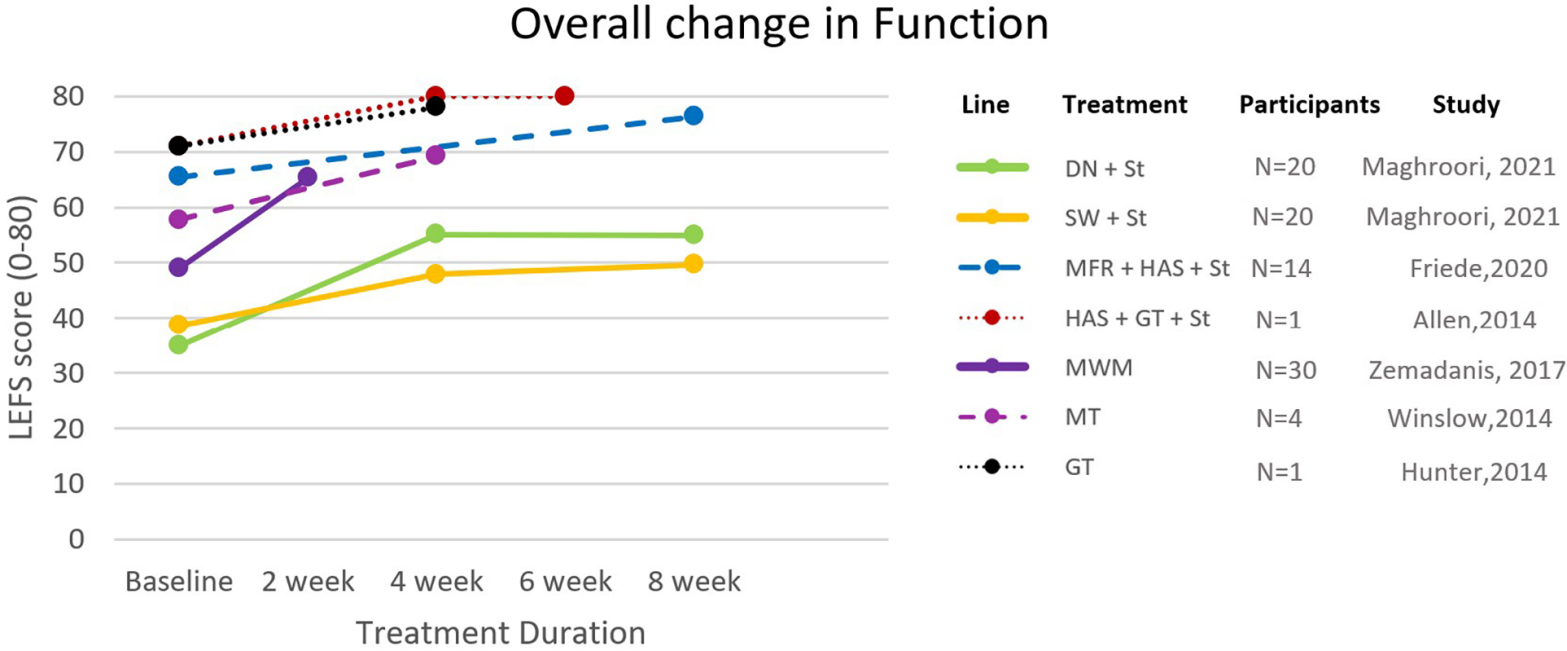
Changes in functional scores across all included studies. Solid lines: RCTs; Dashed lines: Case-control and case series; Dotted lines: Case reports; DN, dry needling; GT, gait retraining; HAS, hip abductor strengthening; MFR, myofascial release; MT, manual therapy; MWM, movement-with-mobilization; St, stretching; SW, shockwave.

The effectiveness of the strategies on pain was assessed by NRPS, Visual Analogue Scale (VAS), and Allan McGavin Health Status Index (AMI); lower extremity function was assessed by Lower Extremity Functional Scale (LEFS) and AMI (Figures 2, 3). Only three studies reported long-term follow-ups (8, 25, 26). Other assessments included hip abduction strength with a handheld dynamometer (15, 27, 28), hip abduction torque (8), and 3D kinematics (29). Nevertheless, a lack of standardization hindered the inclusion of these variables throughout the included articles.

### 3.1 Assessment of risk of bias

Based on the NIH Quality Assessment tool, the mean methodological score was 66%. According to the classification of methodological quality, twelve out of thirteen studies were deemed good quality (50%–75%) and one was deemed excellent quality with a score of 93%. Individual scorings of each study can be found in Tables 1, 2. Detailed information is found in Supplementary Files 1, 2.

**Table 1:** Changes in pain and function in studies using combined treatment strategies.

Study characteristics							Duration of Intervention					Overall
Study (ref)	Treatment strategy	Type of study	Quality NIH (%)	Sample size	Cond.	Outcome measure	Baseline	2 weeks	4 weeks	6 weeks	8 weeks	Outcome
Maghroori et al. (30)	DN + St	RCT	71%	*N* = 20	At rest	Pain	89 ± 11	–	30 ± 21.1	–	17 ± 17.3	↓80%^b^
						Function	34.9 ± 16.6	–	55.1 ± 17.8	–	54.9 ± 17.7	↑57%^b^
	SW + St	RCT	71%	*N* = 20	At rest	Pain	83 ± 18	–	38.2 ± 23.9	–	36 ± 26.7	↓57%^b^
						Function	38.6 ± 12.5	–	47.9 ± 14.3	–	49.7 ± 14	↑29%^b^
Weckström & Söderström (31)	SW + HAS	RCT	71%	*N* = 12	Running	Pain^α^	^a^	–	↓51%	–	↓75%	↓75%^b^
						Function	–	–	–	–	–	–
	MT + HAS	RCT	71%	*N* = 12	Running	Pain^α^	^a^	–	↓61%	–	↓66%	↓66%^b^
						Function	–	–	–	–	–	–
Schwellnus et al. (32)	MT + St + US	RCT	64%	*N* = 20	At rest	Pain	≈75	≈24	–	–	–	↓68%
						Function	–	–	–	–	–	–
Friede et al. (27)	MFR + HAS + St	Case-control	67%	*N* = 14	At rest	Pain	12.8 ± 14.4	–	–	–	4.2 ± 7.4	↓67%^b^
						Function	65.5 ± 6.6	–	–	–	76.3 ± 4.1	↑17%^b^
					Running	Pain	78.2 ± 14.5	–	–	–	16.8 ± 24	↓78%^b^
						Function	–	–	–	–	–	–
Fredericson etl al. (8)	HAS + St + US	Case series	56%	*N* = 24	At rest	Pain	^a^	–	–	^a^	–	↓ND^b^
						Function	–	–	–	–	–	–
Beers et al. (28)	HAS + St + US	Pre-test post-test	67%	*N* = 16	At rest	Pain^β^	11.1/15	13.3/15	13.3/15	13.9/15	–	↓27%
						Function^β^	26.9/30	28.4/30	28.6/30	28.6/30	–	↑10%
Allen (26)	HAS + GT + St	Case report	56%	*N* = 1	At rest	Pain	40	–	0	0	–	↓100%
						Function	71	–	80	80	–	↑13%
Shamus & Shamus (25)	HAS + GT	Case report	67%	*N* = 2	At rest	Pain	85	–	0	–	–	↓100%
						Function	–	–	–	–	–	–

Pain: measured on VAS; function: measured on LEFS; painα: measured on NRPS; painβ: measured on AMI; functionβ: measured on AMI; AMI: Allan McGavin Health Status Index; DN, dry needling; EA, electroacupuncture; GT, gait retraining; HAS, hip abductor strengthening; LEFS, lower extremity functional scale; MFR, myofascial release; MT, manual therapy; MWM, movement-with-mobilization; ND, no reported data; NPRS, numeric pain rating scale; SW, shockwave St, stretching; US, ultrasound; VAS, visual analog scale.

^a^ No reported value.

^b^ Statistically significant result.

**Table 2:** Changes in pain and function in studies using single treatment strategies.

Study characteristics							Duration of Intervention					Overall
Author (year)	Treatment strategy	Type of study	Quality NIH (%)	Sample size	Cond.	Outcome measure	Baseline	2 weeks	4 weeks	6 weeks	8 weeks	Outcome
Passive treatment strategies												
Zemadanis & Bestos (33)	MWM	RCT	93%	*N* = 30	At rest	Pain^α^	51.1 ± 7.2	20 ± 9.1	–	–	–	↓61%^b^
						Function	49 ± 5.8	65.3 ± 4.8	–	–	–	↑33%^b^
Winslow (34)	MT	Case series	67%	*N* = 4	At rest	Pain^α^	85.6 ± 10.4	–	25 ± 44.1	–	^a^	↓71%
						Function	57.7 ± 6.6	–	69.2 ± 11.5	–	^a^	↑20%
Wong (35)	EA	Case report	56%	*N* = 1	At rest	Pain	70	–	–	10	–	↓86%
						Function	–	–	–	–	–	–
Active treatment strategies												
McKay et al. (15)	Multiplanar HAS	RCT	71%	*N* = 8	At rest	Pain^α^	^a^	–	–	–	^a^ ^,^ ^b^	↓ND^b^
						Function	^a^	–	–	–	^a^ ^,^ ^b^	↑ND^b^
	HAS			*N* = 8		Pain^α^	^a^	–	–	–	^a^	↓ND
						Function	^a^	–	–	–	^a^	↑ND
	St			*N* = 8		Pain^α^	^a^	–	–	–	^a^	↓ND
						Function	^a^	–	–	–	^a^	↑ND
Hunter et al. (29)	GT	Case report	67%	*N* = 1	Running	Pain	50	–	0	–	–	↓100%
						Function	71	–	78	–	–	↑10%

Pain: measured on VAS; function: measured on LEFS; Painα: measured on NRPS; EA, electroacupuncture; GT, gait retraining; HAS, hip abductor strengthening; LEFS, Lower extremity functional scale; MT, manual therapy; MWM, movement-with-mobilization; ND: no reported data; NPRS, numeric pain rating scale; VAS, visual analog scale.

^a^ No reported value.

^b^ Statistically significant result.

### 3.2 Combined treatment strategies

A comprehensive overview of all the studies using combined treatment strategies including the study characteristics, treatment interventions, outcome measures (pain and function), duration of intervention, and the overall outcome is presented in Table 1.

Among the eight studies that used combined treatment strategies, six combined active and passive interventions, while two used a combination of only active interventions (Table 1). Combined treatment studies that reported long-term follow-ups were two case reports using gait retraining with HAS and stretching exercises (25, 26) and one case series using HAS and stretching exercises with ultrasound (8). They reported no pain after intervention which was maintained at 4-month (26) and 6-month (8, 25) follow-ups with no recurrence of ITBS.

Six studies used HAS exercises as a primary or adjunct intervention and showed improvements in outcomes (8, 19–21, 31, 32). Two studies utilized shockwave therapy along with stretching or HAS exercises and found statistically significant improvements in outcomes (30, 31). Of these, the study using HAS exercises (31) showed higher improvement than the one using stretching exercises (30) Similar results were obtained with the combination of HAS exercises with manual therapy techniques. The study that combined manual therapy with stretching exercises and ultrasound did not show a statistically significant improvement (32) (Table 1).

### 3.3 Single treatment strategies

A detailed overview of all the studies using single treatment strategies including the study characteristics, treatment interventions, outcome measures (pain and function), duration of intervention, and the overall outcome is presented in Table 2.

Of the five studies using single treatment approaches, three used only passive strategies while two used only active strategies (Table 2). Two studies used manual therapy techniques only and reported improvements in outcomes (33, 34). The improvements in outcomes in the studies using a combination of manual therapy with HAS exercises were higher than in the studies using manual therapy alone (27, 31).

Among the two studies that used only active treatment strategies, the RCT by McKay et al. (15) found that multiplanar HAS exercises resulted in an increased benefit compared to standard HAS or stretching exercises alone. Another study (case report) used only gait retraining and reported improvements in pain and function (29) (Table 2).

## 4 Discussion

The purpose of this systematic review was to provide an overview of the existing literature and to discuss the effects of different conservative treatment strategies on pain relief and function outcomes in runners with ITBS. This review scrutinized thirteen studies based on a range of conservative treatment approaches, of which eight studies used combined treatment strategies while five used single treatment strategies. Overall, hip abductor strengthening exercises were found to form a key component in the conservative treatment of ITBS in runners. Additionally, the heterogeneity in study designs did not allow for a meta-analysis.

The first key finding of this systematic review is the use of HAS exercises as a key component in the treatment of ITBS from the variety of available conservative methods. HAS exercises were used in six out of the eight combined treatment studies (Table 1), and one out of the five single treatment strategies (Table 2). These results indicate that increased hip abductor strength leads to a reduction in pain and an improvement in function in runners with ITBS (8, 19–21, 31, 32). These improvements might be attributed to the increase in hip abductor strength by reducing the biomechanical forces that place added strain on the iliotibial band, making it less susceptible to impingement against the lateral femoral epicondyle during the foot strike of the gait cycle (8, 11). On the one hand, a weakened Gluteus Medius muscle has been reported to lower body impairments and ITBS (8, 15). Hence, a dysfunctional Gluteus Medius might lead to increased hip adduction and internal rotation, which can exacerbate friction and compression of the ITB at the lateral knee, contributing to ITBS (15). On the other hand, the potential benefits of HAS exercises might involve a more active Gluteus Medius that consequently leads to improved strength and function linked with ITBS (7, 15).

This review highlights a variety of HAS exercises frequently used across studies, including side-lying hip abduction, pelvic drops, single-leg squats, modified side planks with hip abduction, and band-resisted side-stepping. Additional exercises mentioned less frequently include forward-backward lunges, clamshells, skater-running man, cook hip lift, glute bridge, and double-leg hip thrust (8, 17, 19–21, 31, 32). Despite this commonality in exercise selection, formulating a standardized HAS protocol presents challenges due to variations in the exercises' frequency, intensity, progression, and the overall duration of the interventions reported. Moreover, the studies diverged in their outcomes concerning pain relief and functional improvement, underscoring the complexity of establishing a one-size-fits-all protocol.

Nevertheless, a progression of HAS training is the implementation of a multiplanar HAS exercise protocol, as promoted by McKay et al. (15). This approach might yield superior outcomes by enhancing muscle activation in the hip abductors more effectively than traditional HAS exercises (15). Empirical evidence from this randomized controlled trial demonstrated that the multiplanar protocol significantly outperformed both stretching and conventional HAS exercises in improving pain and function (15). Despite these promising results, the study's limitation to female runners and a small sample size (eight participants per group after dropouts) suggests that further research with a broader and more diverse participant base is necessary (15). Such research should also address sex-specific known differences in injury diagnosis and rehabilitation options (36), to ensure the applicability and efficacy of HAS exercises across different populations. Additionally, sex differences have been reported with women being twice as likely to develop ITBS than men (1, 37, 38).

A second key finding is a superior efficacy of employing a multimodal approach to treatment strategies over relying on single interventions. The comparative analysis reveals that combined treatment strategies (27, 30, 31), which have been documented to produce statistically significant outcomes (Table 1), achieved an average pain reduction of approximately 71%, in contrast to the 61% reduction observed with single treatment methods (Table 2) (33). This underscores that combining different treatments can provide greater benefits. Such a holistic approach is more congruent with addressing the complex, multifaceted nature of the etiology behind ITBS (2, 10, 12, 13, 15), suggesting that a comprehensive treatment regimen could offer more effective pain management and functional outcomes.

Incorporating HAS exercises alongside various treatment modalities—such as shockwave, manual therapy, myofascial release, stretching, ultrasound, and gait retraining—has shown positive outcomes in reducing pain and enhancing functional capabilities (8, 19–21, 31, 32). This multifaceted approach to treatment not only broadens the scope of intervention strategies but also reinforces the efficacy of combining HAS exercises with other therapeutic techniques. The observed improvements in pain and function from these combinations align with recommendations from previous systematic reviews (6, 18). These reviews advocate for the integration of HAS exercises, stretching routines, and the use of anti-inflammatory medications as a comprehensive treatment plan for individuals suffering from ITBS.

For example, shockwave therapy with HAS exercises demonstrated superior results within 4 weeks than ultrasound with HAS and stretching exercises over 6 weeks (Table 1). These findings can be attributed to the difference in mechanisms of action of the two modalities and are supported by previous studies which showed more pronounced improvements with shockwave therapy compared to ultrasound in overuse injuries (39, 40). Based on this evidence, it might be suggested that HAS exercises with a graded progression for 4 to 8 weeks form the core of conservative treatment in runners with ITBS.

Additionally, different manual therapy techniques were used in combination with active strategies like HAS and stretching exercises. Overall, the effectiveness of manual therapy techniques in runners with ITBS depends on the specific technique utilized and the treatment duration (27, 33). Grounded on the results of the combined studies, the myofascial release technique combined with HAS exercises for 6 weeks was most beneficial in reducing pain and improving function (27). Yet, a longer intervention of 10–12 weeks might lead to better myofascial adaptations of the exercise (15).

Gait retraining emerges as an innovative approach within the array of strategies examined in this review, implemented both as a single intervention (29) and in conjunction with hip abductor strengthening (HAS) exercises (25, 26). The incorporation of gait retraining with HAS exercises represents a dual active treatment strategy that has demonstrated promising outcomes in alleviating symptoms within a timeframe of 4–6 weeks, with these improvements persisting over long-term follow-up periods (31–33). However, the interpretation of these findings requires caution due to the limited scope of the studies, preventing broad generalizations.

A critical examination of gait retraining's efficacy reveals its dependency on several variables, including the specific type of gait modification employed (e.g., adjusting step rate, transitioning to a non-rearfoot strike pattern, or applying a multi-parameter approach), the duration of the injury, and the runner's mileage (16). This complexity highlights the intricate relationship between running biomechanics and the risk of running-related injuries. Therefore, there is a pressing need for further rigorous research to prove the role of gait retraining in the comprehensive treatment regimen for runners afflicted with ITBS, ensuring the approach is both scientifically validated and tailored to individual needs.

From the perspective of intervention duration, the technique of mobilization-with-movement (MWM), a form of manual therapy, demonstrated notable effectiveness within a brief two-week period in a randomized controlled trial involving 30 participants (33). Despite these promising short-term results, the absence of follow-up assessments limits the ability to gauge the long-term benefits of MWM, making such evaluations crucial for a comprehensive understanding of its efficacy. While MWM alone yielded positive outcomes, the studies that integrated manual therapy with HAS exercises led to even greater improvements (27, 31). This finding suggests that a combined approach may offer enhanced benefits for treating ITBS. Therefore, there is a clear need for future research to explore the sustained effectiveness of combining MWM with HAS exercises, aiming to establish a more effective treatment paradigm for ITBS.

Stretching was utilized as a single treatment method in 20% of the cases (15), while it was incorporated into 75% of the combined treatment strategies (8, 26–28, 30, 32), aligning closely with findings from a recent scoping review where stretching was reported in 31% of clinical studies and 78% of review articles (41). Despite its frequent use, the specific study included in this review that investigated stretching in isolation did not quantify its effects on pain relief or functional improvement (15), which constrains a detailed comparison with combined treatment approaches. Moreover, a recent narrative review concluded that the evidence supporting the efficacy of stretching as a conservative treatment for ITBS is limited (42).

Contrastingly, an earlier systematic review highlighted stretching exercises as a beneficial component of a comprehensive treatment plan when combined with manual therapy and ultrasound for ITBS patients (17). It was hypothesized that stretching could potentially elongate the iliotibial band, thereby diminishing strain. Nonetheless, recent contributions to the literature, as seen in the current review, have not corroborated significant changes in iliotibial band length due to stretching (27, 28). Despite this, improvements in pain and functionality were observed, which may be attributed to the inclusion of hip abductor strengthening (HAS) exercises within the combined treatment protocols. A possible explanation might be that HAS exercises activated the Gluteus Medius muscle, leading to improved function and strength (15). These findings underscore the critical role of HAS exercises in effectively managing ITBS in runners, suggesting that their benefits may overshadow the direct impact of stretching on the iliotibial band itself.

Evaluating pain and function specifically during running holds particular significance in the management of ITBS, a condition inherently linked to running activities and characterized by symptoms that intensify with running (43, 44). Such assessments are pivotal for clinicians aiming to devise rehabilitation strategies that not only address the injury but also strategically support a runner's safe and effective return to their sport. Within the scope of this review, only three studies assessed pain and function during running (27, 29, 31). Notably, gait retraining emerged as the most effective single treatment approach, achieving complete elimination of pain during running, as documented in one of the studies (29). This was closely followed by a combined treatment strategy involving myofascial release, HAS exercises, and stretching, which reported a substantial 78% reduction in pain (27). Yet, the robustness of these results must be considered with caution since the small scale of the studies in question—one being a case-control study with 14 participants (27) and the other is a case report focusing on a single individual. These limitations emphasize the need for broader research to firmly establish the efficacy of these treatments within the context of ITBS rehabilitation.

This systematic review demonstrates that ITBS can be managed with conservative treatment modalities. This aligns with recent findings showing that conservative therapy can significantly reduce ITBS symptoms (45). The reduction in pain and improvement in function with treatments that include hip abductor strengthening (HAS) are consistent with the structural and functional benefits observed from strengthening the hip muscles, similar to those reported in managing patellofemoral pain (46). Is it shown that strengthening the hip abductors in ITB patients leads to higher strength in objective testing, explaining the positive effect of pain release (15). Additionally, while the exact mechanisms of action for other conservative treatments such as shockwave therapy, dry needling, and stretching remain unclear, these methods have also shown benefits in managing ITBS (30, 42, 47). However, the exact mechanisms of action behind are widely unknown. It has been speculated that eccentric stress and soft-tissue mobilization induce an upregulation of the collagen synthesis and breaks of micro adhesions leading to a re-organization of collagen fiber morphology as well as a reduction of pain-generating substance P positive nerve endings (34, 48). Similarly, shockwave therapy might act through the promotion of cellular proliferation and pain modulation (33, 47). Further research investigating these mechanisms of action should support or disregard these theories.

### 4.1 Limitations

Although limiting the study type to RCTs would have increased the level of evidence of the present review, the scarcity of available RCTs on this topic necessitated the inclusion of other study designs to ensure a comprehensive review. Moreover, the inclusion of diverse study designs provides a more holistic view of the current state of research and helps identify gaps for future high-quality RCTs. Further, differences in treatment durations and treatment strategies used across the studies did not allow for a direct comparison between studies. The heterogeneity in study designs, treatment durations, outcome measures employed, participant characteristics, and interventions used hinder the ability to draw firm conclusions on the management of ITBS.

Additionally, due to the lack of specification in the included studies, the results of this review did not account for the stage of the injury (acute, sub-acute, or chronic), which can be an influencing factor in the effectiveness of treatment strategies (49). Also, most included studies did not specify the activity limitations implemented during the treatment intervention which can also have an impact on the outcomes. Moreover, the heterogeneity of samples across the included studies led to a wide variation in baseline measurements as seen in Figures 2, 3, and must be considered while interpreting the results.

From the risk of bias perspective, the current systematic review is limited to the methodological rigor of each included study. The outcome parameters in this review were assessed mainly with self-reported scales, i.e., pain via VAS, NPRS, AMI, and function via LEFS, AMI. Therefore, caution should be considered in clinical settings, and proper instructions are recommended to assess pain and function in patients with ITBS. A reduced number of studies included other measurements such as hip abduction strength via a handheld dynamometer, but a lack of standardization limited the comparison among the studies. Additionally, future research should focus on a combination of conservative treatments including clear outcome measures, the severity of ITBS, and a holistic approach towards its biomechanics, physiological, and psychological aspects from the multifactorial nature of running-related injuries.

### 4.2 Conclusion

In conclusion, HAS exercises, particularly when integrated with shockwave or manual therapy, are effective in reducing pain and improving function in runners with ITBS. Other methods appear to be promising, however, the current literature on gait retraining and other strategies lacks comprehensive, high-quality studies. Addressing these gaps could refine ITBS management, enhancing treatment outcomes and facilitating runners’ return to sport.

## Data Availability

The original contributions presented in the study are included in the article/Supplementary Material, further inquiries can be directed to the corresponding author.
